# Comment on “Recent Advance in Source, Property, Differentiation, and Applications of Infrapatellar Fat Pad Adipose-Derived Stem Cells”

**DOI:** 10.1155/2021/9824616

**Published:** 2021-04-13

**Authors:** Elena Stocco, Andrea Porzionato, Raffaele De Caro, Veronica Macchi

**Affiliations:** ^1^Department of Neurosciences, Institute of Human Anatomy, University of Padova, Padova, Italy; ^2^L.i.f.e.L.a.b. Program, Consorzio per la Ricerca Sanitaria (CORIS), Veneto Region, Padova, Italy

A review article recently published by Zhong et al. [1] interestingly considered the knee adipose tissue infrapatellar fat pad (IPFP) as a significant reservoir of mesenchymal stem cells (MSCs).

The MSCs from bone marrow, synovium, and subcutaneous fat have long been suggested for vanguard therapies in osteoarthritis (OA). Specific immunophenotypic characteristics (allowing for differentiation towards the chondrogenic/osteogenic/adipogenic lineages), together with peculiar immunomodulatory properties, have made the MSCs a valuable resource in tissue engineering and regenerative medicine approaches. However, the need for a second damage site for their isolation has prompted the search for viable alternatives. In this framework, the review analysis by Zhong et al. [1] stands out, clearly outlining the recent interest of the scientific community for the IPFP as a promising supplier of MSCs.

To date, there is a certain growing consensus in identifying the IPFP and the synovial membrane as a unique anatomofunctional unit that actively communicates and interacts with the surrounding joint environment through molecular signals also displaying a key role in the onset and progression of OA [2, 3]. In a clinical practice, when conservative strategies are shown to not be effective in the relief of OA painful symptoms, knee surgery with synovial membrane and IPFP resection (partial or total) may be scheduled [4]. The chance to take therapeutic advantage from a waste tissue (i.e., IPFP) would be highly rewarding: it may ensure gains from tissue engineering strategies, avoiding a second surgery site and the eventual related issues. However, according to a recent study by our group [5], the experimental evidence suggests that caution is necessary in claiming the IPFP from OA patients as a high-quality source of stem cells for regenerative purposes. OA is commonly referred to as an age-related disease, but people suffering from OA are more likely to have coexistence of other chronic inflammatory conditions. Among these, obesity and concomitant disorders (e.g., hypertension and type 2 diabetes) stand out, depicting a chronic-stress clinical setting with an increase in oxidative stress mediators (i.e., reactive oxygen species) and accumulation of unfolded proteins [6, 7]. In the knee joint, this results in distress for residents' cellular elements, including OA-IPFP stem cells. Zhong et al. [1] correctly pointed out for the IPFP stem cells a probable reduction in chondrogenic differentiation potential induced by the OA environment; however, our in vitro data [3] also advocated that the ultrastructural, immunophenotypic, and functional characteristics of the OA-IPFP stem cells can be affected.

As reported in Stocco et al. [5], the OA-IPFP stem cells we isolated were characterized by a distinctive immunophenotype (CD73^+^/CD39^+^/CD90^+^/CD105^+^/CD44^–/+^/CD45^–^), a high grade of stemness (*STAT3*, *NOTCH1*, *c-Myc*, *OCT-4*, *KLF4*, and *NANOG*), and self-renewal potential and responsiveness (CD44, CD105, VEGFR2, FGFR2, IL1R, and IL6R) to the microenvironmental stimuli. Moreover, in vitro studies and ultrastructural transmission electron microscopy analyses also showed high metabolic activity and other peculiar characteristics for the OA-IPFP stem cells, suggesting a strong correlation between (a) inflamed environment, (b) cellular-specific features, and (c) IPFP tissue morphology.

The expression of HLA-DR, CD34, Fas, and FasL corroborated for the OA-IPFP stem cells a possible phenotypic reprograming induced by chronic inflammatory conditions, while their response under mechanical stimuli (also verified by the significant expression of the *cortactin* gene) together with the high expression level of the *COL1A1* gene suggested their possible protective-fibrogenic response against mechanical overloading. Interestingly, this evidence supports previous histological findings on IPFP tissue which, in OA, is characterized by highly vascularized lobuli separated by thick fibrous septa [8, 9]. Conversely, the null-expression of *CD38/NADase* (commonly upregulated in OA to reduce glycolytic/mitochondrial metabolism) advocated for OA-IPFP stem cells' inability to counteract NAD^+^-mediated OA inflammation. Thus, inflammation is likely responsible for stress in OA-IPFP stem cells, as also verified by their significant expression of the *calreticulin* gene. In fact, despite the typical anti-inflammatory signature mediated by CD39 and CD73, the OA-IPFP stem cells both showed a limited capacity for controlling the immune response to OA and an adaptive behaviour according to the stress/stimuli they sense (further correlating with the OA-IPFP histological features).

As clearly stated by Zhong et al. [1], IPFP tissue is an interesting stem cell source; however, in cases of inflammation/stress, more investigations are required for an extensive understanding of IPFP stem cells' regenerative potential. In addition to more efforts towards the identification of a shared and clearly effective isolation/culture method, future directions may focus on a full comprehension of the IPFP stem cell behaviour/role in OA also correlating with the OA grade. This approach may help to provide a broad description of OA disease onset and progression and also provides strong evidence to help in the identification of vanguard effective treatments.

## References

[1] Zhong Y. C., Wang S. C., Han Y. H., Wen Y. (2020). Recent advance in source, property, differentiation, and applications of infrapatellar fat pad adipose-derived stem cells. *Stem Cells International*.

[2] Macchi V., Stocco E., Stecco C. (2018). The infrapatellar fat pad and the synovial membrane: an anatomo-functional unit. *Journal of Anatomy*.

[3] Belluzzi E., Stocco E., Pozzuoli A. (2019). Contribution of infrapatellar fat pad and synovial membrane to knee osteoarthritis pain. *BioMed Research International*.

[4] Kim Y. M., Joo Y. B. (2019). Arthroscopic treatment of infrapatellar fat pad impingement between the patella and femoral trochlea: comparison of the clinical outcomes of partial and subtotal resection. *Knee surgery & related research*.

[5] Stocco E., Barbon S., Piccione M. (2019). Infrapatellar fat pad stem cells responsiveness to microenvironment in osteoarthritis: from morphology to function. *Frontiers in Cell and Development Biology*.

[6] Courties A., Sellam J., Berenbaum F. (2017). Metabolic syndrome-associated osteoarthritis. *Current Opinion in Rheumatology*.

[7] Courties A., Berenbaum F., Sellam J. (2019). The phenotypic approach to osteoarthritis: a look at metabolic syndrome- associated osteoarthritis. *Joint, Bone, Spine*.

[8] Favero M., el-Hadi H., Belluzzi E. (2017). Infrapatellar fat pad features in osteoarthritis: a histopathological and molecular study. *Rheumatology*.

[9] Fontanella C. G., Carniel E. L., Frigo A. (2017). Investigation of biomechanical response of Hoffa's fat pad and comparative characterization. *Journal of the Mechanical Behavior of Biomedical Materials*.

